# Psychological Impact of parental cancer on children

**DOI:** 10.1192/j.eurpsy.2022.1070

**Published:** 2022-09-01

**Authors:** D. Falfel, S. Korbi, Y. Berrazaga, N. Mejri, W. Homri, H. Boussen, R. Labbane

**Affiliations:** 1 Razi hospital, Psychiatry Ward, Manouba, Tunisia; 2 Abderahmen mami hospital, Oncology Ward, Ariana, Tunisia; 3 Razi Hospital, Psychiatry Ward, Manouba, Tunisia

**Keywords:** behavior, coping, Children, parental cancer

## Abstract

**Introduction:**

Cancer is often a diagnosis that generates instability in many Tunisian families. Children of parents with cancer may respond differently to the treatment. Communication about cancer in Tunisian families needs sometimes professional intervention mainly with children.

**Objectives:**

We aimed to assess psychological impact of cancer parents on their children.

**Methods:**

We interviewed 103 parents of children aged 6-18 years between July and December 2020. Children were not interviewed as they were not allowed into the chemotherapy treatment rooms. The questionnaire included items about emotional and behavioral impact on children.

**Results:**

Patients’ characteristics are are shown in Table 1. In our study, 85 patients (82.5%) told their children they were « sick ». Among the children who were not aware of their parent’s condition, there were significantly more preschoolers, p=0.001. The reasons given by the parents in these cases were the young age of their children (60%) and the fear of generating emotional and behavioral trauma and threatening their psychosocial equilibrium (40%). In our participants 88.3% reported communication disorders with their children when referring to the parental illness.

**Conclusions:**

Parental cancer may have unexpected consequences on children’s behavior which should be handled by a specialist , hence efforts should be made for early detection and better understanding of these disorders.

**Disclosure:**

No significant relationships.

